# A Review of Events That Expose Children to Elemental Mercury in the United States

**DOI:** 10.1289/ehp.0800337

**Published:** 2009-01-12

**Authors:** Robin Lee, Dan Middleton, Kathleen Caldwell, Steve Dearwent, Steven Jones, Brian Lewis, Carolyn Monteilh, Mary Ellen Mortensen, Richard Nickle, Kenneth Orloff, Meghan Reger, John Risher, Helen Schurz Rogers, Michelle Watters

**Affiliations:** 1 Agency for Toxic Substances and Disease Registry, Atlanta, Georgia, USA; 2 Centers for Disease Control and Prevention, Atlanta, Georgia, USA; 3 EDS, an HP Company, Plano, Texas, USA; 4 TKC Integration Services, LLC, Anchorage, Alaska, USA

**Keywords:** children, elemental mercury, environmental health, exposure, United States

## Abstract

**Objective:**

Concern for children exposed to elemental mercury prompted the Agency for Toxic Substances and Disease Registry and the Centers for Disease Control and Prevention to review the sources of elemental mercury exposures in children, describe the location and proportion of children affected, and make recommendations on how to prevent these exposures. In this review, we excluded mercury exposures from coal-burning facilities, dental amalgams, fish consumption, medical waste incinerators, or thimerosal-containing vaccines.

**Data sources:**

We reviewed federal, state, and regional programs with information on mercury releases along with published reports of children exposed to elemental mercury in the United States. We selected all mercury-related events that were documented to expose (or potentially expose) children. We then explored event characteristics (i.e., the exposure source, location).

**Data synthesis:**

Primary exposure locations were at home, at school, and at other locations such as industrial property not adequately remediated or medical facilities. Exposure to small spills from broken thermometers was the most common scenario; however, reports of such exposures are declining.

**Discussion and conclusions:**

Childhood exposures to elemental mercury often result from inappropriate handling or cleanup of spilled mercury. The information reviewed suggests that most releases do not lead to demonstrable harm if the exposure period is short and the mercury is properly cleaned up.

**Recommendations:**

Primary prevention should include health education and policy initiatives. For larger spills, better coordination among existing surveillance systems would assist in understanding the risk factors and in developing effective prevention efforts.

Mercury occurs naturally in the earth’s crust. It exists in the environment as the result of natural processes and human activities. Mercury forms a dense, silvery liquid at room temperature (density = 13.534 g/cm^3^). Liquid mercury has a relatively low vapor pressure (0.0085 mmHg at 25°C) and volatilizes slowly at room temperature. If not managed properly, indoor mercury spills can release mercury into the air over weeks or even years [Agency for Toxic Substances and Disease Registry (ATSDR) 1999]. Heating mercury results in much higher, potentially lethal airborne mercury concentrations, especially in indoor spaces (Putman and Madden 1972; Solis et al. 2000; Taueg et al. 1992).

Although mercury may be ingested, it is poorly absorbed in the normal gastrointestinal tract [World Health Organization (WHO) 1991]. Dermal absorption of mercury is also a minor exposure pathway (Hursh et al. 1989). However, mercury vapor is readily absorbed by the lungs, making inhalation the exposure route of greatest concern. The ATSDR minimal risk level for chronic mercury inhalation is 0.2 μg/m^3^ (ATSDR 1999).

## Health concerns

The health effects associated with acute elemental mercury exposure vary with the magnitude and duration of exposure. The potential health effects from inhaling high mercury concentrations (e.g., ~ 10,000 μg/m^3^) are primarily respiratory [ATSDR 1999; U.S. Environmental Protection Agency (EPA) 2002] and include pneumonitis, bronchiolitis, pulmonary edema, and in some instances death. Exposure to mercury vapor (e.g., 10–100 μg/m^3^) over prolonged time periods can cause neurobehavioral effects, mood changes, and tremors. Chronic exposures are associated with hypertension and autonomic nervous system dysfunction (WHO 2003). Mercury exposure is also associated with acrodynia (painful extremities), a rare hypersensitivity reaction to mercury (Caravati et al. 2008).

Children are more sensitive to mercury and are at greater risk than adults after certain mercury exposures (ATSDR 1999; Rogers et al. 2007). Urine mercury levels < 5 μg/L urine have not been associated with neuro-cognitive effects in children (Bellinger et al. 2006; DeRouen et al. 2006).

## Reference levels

Mean [95% confidence interval (CI)] urine mercury levels in the 2003–2004 Centers for Disease Control and Prevention (CDC) National Health and Nutrition Examination Survey (NHANES) were 0.254 μg/L (95% CI, 0.213–0.304 μg/L) for children 6–11 years of age and 0.358 μg/L (95% CI, 0.313–0.408 μg/L) for children 12–19 years of age (CDC 2005c, 2007) (Table 1). In 2001–2002, the mean NHANES total blood mercury levels in children 1–5 years of age was 0.32 μg/L (95% CI, 0.27–0.38 μg/L), and the 95th percentile was 1.2 μg/L (95% CI, 0.9–1.6 μg/L) (CDC 2005c).

## Objective

In New Jersey, an industrial building formerly used to manufacture mercury thermometers was renovated and converted in 2004 to a child care facility (ATSDR 2007b). Unfortunately, the property was not adequately cleaned before renovation, leaving residual contamination with elemental mercury (ATSDR 2007b). Such contamination can cause significant exposure for children or adults who are present. People exposed in these types of events may require medical evaluation and biomonitoring.

Concern for children exposed to elemental mercury through inhalation prompted the ATSDR and the CDC to review existing data to identify the common sources of elemental mercury exposure in children; describe the location, demographics, and proportion of children affected by elemental mercury exposure events in the United States; and make recommendations on preventing elemental mercury exposures and responding appropriately to spills and releases.

This review does not focus on mercury-related health effects or treatment, nor does it review mercury exposures associated with coal-burning facilities, dental amalgams, fish consumption, medical waste incinerators, or thimerosal-containing vaccines. Unless other wise stated, mercury refers to elemental mercury.

## Methods

We conducted a comprehensive review of the existing exposure data sources and the scientific literature to identify and quantify common sources of mercury exposure for children in the United States and to describe the location, demographics, and proportion of children affected by such exposures. We also reviewed numerous mercury exposure prevention initiatives.

### Existing exposure data sources

The data sources reviewed included an extensive list of federal, state, and regional programs that capture information on spills and other hazardous releases. We identified and contacted key personnel to assess the relevance of the data. The following five data sources contained relevant data: ATSDR health consultations (HCs) and emergency response calls, ATSDR Hazardous Substances Emergency Events Surveillance (HSEES), U.S. Coast Guard National Response Center database, American Association of Poison Control Centers (AAPCC) National Poison Data System, and Association of Occupational and Environmental Clinics (AOEC) Pediatric Environmental Health Specialty Units (PEHSUs).

We considered other data sources, such as those maintained by the U.S. EPA and the National Institute for Occupational Safety and Health, but they did not contain information relevant to this report.

We used the following criteria to select relevant mercury releases (exposure events): First, the event had to be reported between 2002 and 2007, a time frame that represents the most current information available on exposure events. Second, the event had to take place in the United States. Finally, the event had to expose or potentially affect a child (or children) ≤ 18 years of age. If the data source did not contain information on the age of the exposed or affected persons, the event was included if it occurred at a location frequented by children (e.g., an elementary or secondary school, a child care facility, or a private residence). These criteria were treated as guidelines, because each data source has its own functionality and limitations on how events could be queried. We provide a description of the information available below, by data source.

### Literature review

We supplemented the information from the data sources by searching the National Library of Medicine’s PubMed (National Library of Medicine 2008) and Thomson Scientific’s Web of Science (Thomson Reuters 2008) for mercury exposure events involving children ≤ 18 years of age. We used the search terms “elemental mercury,” “metallic mercury,” and “liquid mercury.” We reviewed only publications documenting U.S.-based exposures between January 2002 and December 2007 and omitted publications that did not describe a specific exposure event.

### Exposure scenarios

To further characterize elemental mercury exposure events and locations, we present several common exposure scenarios, to broadly illustrate the nature and public health impact of such events.

### Initiatives for preventing mercury exposure

We reviewed a selection of ongoing federal- and state-based mercury initiatives to highlight innovative and successful approaches to reducing childhood mercury exposure.

## Data Synthesis

### Existing exposure data sources

#### ATSDR HCs and emergency response calls

ATSDR is the lead federal public health agency for implementing the health provisions of the Comprehensive Environmental Response, Compensation, and Liability Act of 1980 (“Superfund”) and its amendments. Under this act, ATSDR evaluates the public health impact of hazardous substances released into the environment. ATSDR receives numerous inquiries regarding mercury exposure events. Although some inquiries are not systematically recorded, some are documented as ATSDR HCs, and others are documented as emergency response calls.

We reviewed the HCs to identify events that document potential mercury exposure to children. We selected events if there was a completed mercury exposure pathway in air and children were potentially exposed.

During the years 2002–2007, ATSDR and its state cooperative agreement partners produced HCs for 26 events that exposed or potentially exposed children to elemental mercury in air. These events took place between 2001 and 2006. Of these 26 incidents, two children were potentially exposed in more than one location. Fourteen of the 26 (54%) incidents were classified as public health hazards. Although not always mutually exclusive, the location of the exposure event was most frequently described as a home (46%; 12 of 26) or school (42%; 11 of 26). Two of the 26 events (8%) occurred at medical care facilities, one at a child care center (4%), and one in a car (4%). The source of these mercury exposures included use or storage in schools, release from broken thermometers or sphygmomanometers, off-gassing from flooring containing a mercury catalyst, and an unknown source.

The estimated amount of mercury reported in these 26 exposure events ranged from 9 to 700 mL. The maximum indoor air concentrations of mercury ranged from 0.05 μg/m^3^ to > 92 μg/m^3^. Biomonitoring was conducted for children considered exposed in 11 events. The mercury concentrations in blood ranged from below the level of detection (LOD) to 29 μg/L. The urine concentrations ranged from below the LOD to 18 μg/g creatinine. The LOD varied by event. The approximate time interval between exposure and urine collection for testing ranged from 6 to 20 days.

In addition to these HCs, emergency response calls are received from state and local health officials, environmental officials, health care providers, and the general public. From 2000 to 2007, emergency response staff responded to more than 3,000 such inquiries, 459 of which concerned mercury events. The majority of the events occurred in residential settings (44%; 203 of 459) or in schools (13%; 60 of 459). These calls were most often made by private citizens (31%; 143 of 459); many calls concerned cleaning up mercury-related spills (38%; 175 of 459) or health-related questions about mercury exposure (35%; 159 of 459).

Given the relatively few mercury events documented by ATSDR HCs (*n* = 26) compared with the number of mercury-related calls to ATSDR’s emergency response staff (*n* = 459), the HCs represent only a small fraction of all such exposures and may not be representative of mercury events nationwide.

#### ATSDR HSEES

ATSDR developed the HSEES system to collect data on uncontrolled and/or illegal releases of any hazardous substance (ATSDR 2007a). Releases of chemicals for > 72 hr are considered chronic releases and are not captured by HSEES.

Fourteen state health departments report chemical releases to HSEES. The data collected include the type of release, the amount of chemical(s) released, the location of the event (e.g., private residence, school), information about any persons with symptoms or injuries (“victims”), and any possible contributing causes that are known. The number of persons exposed during a chemical release is not captured directly in HSEES. However, using victim data and additional information recorded as optional text, one can estimate the number of exposed persons.

The contributing causes for the release of mercury are categorized as equipment failure, human error, intentional or illegal release, and unknown cause. The human error category includes breaking of or dropping thermometers or other mercury-containing devices or equipment. Intentional or illegal releases include events in which children reportedly played with mercury.

We included the HSEES events from 2002 through 2006 in this compilation if children were potentially exposed to elemental mercury (ATSDR HSEES, unpublished data). In HSEES, children are defined as persons ≤ 19 years of age. We omitted events in which releases were only threatened. We selected events if they took place at a private residence, at an elementary or secondary school, or at another location for which children were documented as possibly exposed, injured, or having symptoms associated with mercury exposure.

The HSEES database contained 843 mercury events from 41,709 total events in which hazardous substances were reported to be released from January 2002 through December 2006 (Table 2). Mercury was the only toxicant released in 824 of these events; the remaining 19 mercury events included the release of at least one other hazardous substance. Approximately half of the total mercury events identified (*n* = 409) were classified as potentially exposing children. All 409 events potentially affecting children were mercury-only events.

These events were reported from 17 states; only 12 states participated during the entire time period from 2002 through 2006. The remaining states participated for either 2 or 4 years.

The 409 events potentially affecting children were most frequently classified as non-volatilization or spill-only events (88%; 360 of 409). Volatilization of mercury was noted in 6 of the 409 events (2%) as air only and in 40 events (10%) as combined spill and air releases. A fire was noted in one of the 409 events (< 1%). Although liquid mercury has a relatively low vapor pressure and volatilizes slowly at room temperature, some volatilization was likely in some or all of the events described as spill only. Mercury events occurred most frequently in private households (75%; 307 of 409). The most frequent contributing cause of the event was human error (87%; 357 of 409).

Evacuations were ordered in 68 of the 409 events (17%). The median number evacuated per event was 20 people, with a range from 1 to 1,505 people. The total number of people exposed during these 409 events was not captured in HSEES, although 21 people were injured or had symptoms related to an event.

Among the 21 individuals affected, 10 were children. HSEES identified 7 of these children as having specific health effects: gastrointestinal problems (*n* = 3), eye irritation (*n* = 2), respiratory irritation (*n* = 1), and trauma (*n* = 1), although these effects may not be mercury related. In addition, 5 children had elevated levels of mercury in blood/urine (data not shown). Mercury biomarkers are not routinely reported to HSEES, and the prevalence of elevated blood/urine levels is probably underreported.

Limitations do exist in using HSEES data to identify elemental mercury exposures among children. The HSEES data source is intended to build capacity in state health departments for surveillance of acute releases of hazardous substances and to initiate or improve appropriate prevention activities. HSEES was not designed to enumerate and characterize mercury exposure events affecting children. Information on age is captured in HSEES only if the person reports a symptom or requires medical follow-up; for this reason, HSEES data are likely to underestimate the number of children exposed. The magnitude of exposure is difficult to determine because the amount of mercury released or spilled is often reported as a range rather than a specific quantity. Therefore, a reliable calculation of the average amount of mercury released is not possible. Also, the reporting of mercury-related events to HSEES is uneven across the participating states. States with mercury exposure prevention initiatives may report more mercury-related events than do states without mercury initiatives (Michigan Department of Environmental Quality 2007; Minnesota Pollution Control Agency 2006). Lastly, HSEES data cover acute releases only; incidents in which mercury exposure continued for an extended period of time are not included.

#### U.S. Coast Guard National Response Center database

Under federal law, the release or spill of 1 lb (33 mL, ~ 2 tablespoons) or more of mercury into the environment is to be reported to the federal government (U.S. EPA 1989). The primary contact for reporting these events is the National Response Center, operated by the U.S. Coast Guard for the National Response Team established under the National Contingency Plan for Oil and Hazardous Substances Releases (U.S. Coast Guard National Response Center 2002; U.S. EPA 1994).

The National Response Center receives between 25,000 and 30,000 reports of pollution incidents and response drills each year. To identify events for this report, we downloaded data for the years 2002–2007 from the National Response Center Web site and queried them using SAS statistical software (version 9.1; SAS Institute Inc., Cary, NC). We identified mercury-related events by *a*) a Chemical Abstracts Service registry number recorded as “007439-97-6” (denoting mercury was released) or *b*) the word “mercury” reported in the name of the material released, the description of the incident, the description of remedial actions, or the additional information provided. A total of 825 events met this definition between 2002 and 2007. Some exposure events may have taken place before the year in which they were reported.

To assess the number of events in which children were potentially exposed, we conducted two additional searches on the 825 mercury events. First, we always selected school and child care settings as locations where children were potentially exposed by searching for the terms “school” or “daycare” in the fields for incident description, location of the incident, and additional information. Second, we queried the description of the incident and the additional information fields for a series of 10 words or parts of words that represent terms commonly used to describe children (child, kids, infant, baby, teen, toddler, adolescent, boy, girl, student). Of the mercury incidents reported over the 6-year period, 113 (14%; 113 of 825) were events in which children were potentially exposed.

The location of the incident was not reported in 45 (40%) of the 113 events in which children were potentially exposed. When only a street address was given, we used the category “other” to describe the event location. A few events noted more than one exposure location (Table 3).

To compare the amounts of mercury released in different events, we expressed the quantity as milliliters of mercury. The amount of mercury released varied from < 1 mL to approximately 1,900 mL. For example, a fire occurred in one event that released approximately 200 mL of mercury at a school. No information was provided on whether children were present during the release.

Among the 113 events that potentially exposed children, five people were injured, and five people were hospitalized. Whether the five persons injured were the same five persons who were hospitalized is unclear. The states reporting the most incidents that potentially exposed children were Kentucky, Michigan, Mississippi, and Ohio. In 27 events, persons were evacuated. These evacuations took place in a number of locations, including homes and schools.

National Response Center reports contain the initial conditions of each event and are self-reported, often by the spiller. Details often are not known or not volunteered in these initial reports, which results in reporting errors and missing information. On the other hand, mercury spills that draw media attention and state-based mercury initiatives may result in increased and more thorough reporting.

The type of mercury is not systematically recorded, leading to potential misclassification of elemental mercury exposures. Because the National Response Center does not systematically collect the age of persons exposed, the information on children was present only when volunteered. Any analysis of these events is limited by these factors.

#### AAPCC National Poison Data System

The National Poison Data System represents information uploaded in near real time from 61 of 62 U.S. poison control centers. Reporting is passive and voluntary, occurring when a caller reports a known or suspected chemical exposure. Poison control center specialists collect basic demographic data, information about the chemical agent and exposure route, and any reported clinical effects associated with the case. Depending on the nature of the call, a specialist chooses from a preestablished list of chemical agents and selects signs and symptoms from a list of 131 clinical effects. AAPCC classifies persons 19 years of age and younger as children.

**Figure 1:**
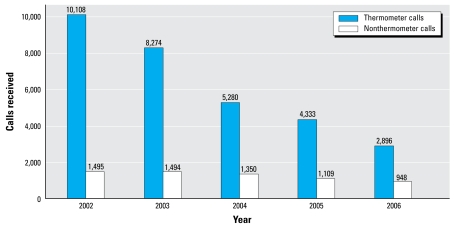
Number of elemental mercury thermometer-related and non-thermometer-related calls concerning children (≤ 19 years of age) made to AAPCC poison control centers, by year: 2002–2006. Thermometer-related calls include general formulation, baby rectal, basal, high/low, oral fever, yellow back glass, and mercury metal thermometers. Non-thermometer-related calls do not include calls regarding amalgams or thermometers.

Between 2002 and 2006, 15,739 mercury-related calls not associated with broken thermometers were made (Figure 1). Most of these calls concerned elemental mercury exposure events (91%; 14,378 of 15,739). The calls concerning children (*n* = 6,396) made up 44% (6,396 of 14,378) of the elemental mercury calls (Figure 1). Although many calls specified dermal exposure or ingestion, such exposures probably also included the potential for inhalational exposure. Michigan and Illinois recorded the most calls to poison control centers for potential childhood mercury exposures. Between 2002 and 2005, 93% or more of the non-thermometer-related mercury exposures in children were coded as an unintentional exposure. In 2006, the percentage of unintentional exposures dropped to 80% (758 of 948). The decrease appears to reflect a single event that prompted 157 calls about an intentional exposure.

Poison control centers also receive a large number of calls regarding broken mercury thermometers. The types of mercury thermometers recorded include general formulation, basal, high/low, oral fever, baby rectal, yellow back glass, and mercury metal. Calls concerning children made up 68% (30,891 of 45,232) of the mercury thermometer calls. Since 2002, the calls related to mercury thermometer exposures have continued to decrease (Figure 1).

AAPCC also records the anticipated health effects of the exposure. Effects are categorized as minor, moderate, major, not followed, and unable to follow (Bronstein et al. 2007). AAPCC describes minor effects as those that have minimally bothersome symptoms and generally resolve rapidly. Moderate effects are more pronounced or more systemic in nature. Major effects are those that may be life threatening or result in disability or disfigurement. Calls are not followed up when the exposure was judged by the AAPCC to be minimal to nontoxic in nature, the amount of the contaminant released was insignificant, or the route of exposure was unlikely to result in a clinical effect. Between 2002 and 2006, most non-thermometer-related (93%; 5,966 of 6,396) and thermometer-related (98%; 30,287 of 30,891) calls were reported as not followed up. Five of the 6,396 calls (<1%) regarding children were about events likely to have had a major effect. All five calls were non-thermometer-related. No major effects were reported among mercury thermometer–related calls.

A strength of the AAPCC data is that calls are classified as those representing an actual human exposure event or classified as other calls, such as those seeking only information. The limitations of the data relate to the passive and incomplete nature of the reporting and the general lack of environmental or human exposure monitoring. In addition, the number of calls that report separate exposure events is unclear; for example, a school-based exposure may prompt a number of concerned parents to call a poison control center. Media attention regarding a mercury exposure event and state-based mercury initiatives probably influences public awareness and the reporting of mercury events to poison control centers.

#### AOEC PEHSUs

The AOEC maintains the PEHSU network to provide consultation to health care professionals and parents for environmental health concerns affecting children and their families (AOEC 2006). Eleven of the 13 PEHSU clinics are located in the United States.

PEHSU consultation data dating before 2004 are not easily queried. Therefore, we queried only events recorded for the period from April 2004 through September 2007. The database does not differentiate among calls about elemental, inorganic, and organic mercury. The database includes age, sex, date of call, and PEHSU region. Of the 242 mercury exposure calls, 120 (50%) concerned potentially exposed boys, 93 (38%) concerned girls, and the sex of the remaining 29 (12%) was not identified. The age of the child was recorded for 225 calls; most of these calls concerned children < 7 years of age. The larger percentage of calls concerning younger children may result from the PEHSU focus on young children.

Since April 2006, the database also has included the role of the caller (e.g., parent, physician) and the exposure location, identified as child care, home, public area, school, waste site, or unknown. PEHSUs received 145 calls during the 18-month period from April 2006 through September 2007. In 108 of the 145 calls (74%), the parent of the potentially exposed child made the call. The most common exposure locations identified were homes and child care facilities.

These data are limited by passive and incomplete reporting and the general lack of environmental or human exposure monitoring data. In addition, how many of these calls may pertain to the same event is unclear. Media attention regarding a mercury exposure event and the implementation of state-based mercury initiatives are likely to influence public awareness and the reporting of mercury events to PEHSUs.

### Literature review

Ten published reports met the criteria for inclusion (Azziz-Baumgartner et al. 2007; Baughman 2006; CDC 2005a, 2005b; Cherry et al. 2002; Gattineni et al. 2007; Gordon 2004; Hryhorczuk et al. 2006; Johnson 2004; Tominack et al. 2002).

These 10 publications reported 13 events that exposed approximately 1,393 children between 1998 and 2004. The year of the exposure was not reported for two of these events. The children exposed ranged from 2 to 18 years of age. Exposures took place in homes, cars, schools, and school buses. In eight events, a child obtained mercury by stealing it. Mercury was stolen from a school in 6 of the 13 events (46%), once from a dental office (8%), and once from an industrial site (8%). The mercury was subsequently dispersed or sold to other children. When reported, the estimated amount of mercury spilled/released ranged from 9 to 701 mL. The events reporting the largest releases typically occurred after children stole mercury from an industrial site (~ 701 mL mercury released) or a school (30–40 mL mercury released). When mercury was taken from a school, children typically played with the material at school and then at home, producing exposures in multiple locations.

In four additional reports, the exposure resulted from mercury found in the home. The sources of mercury included mercury-containing devices, prior spills, and mercury stored in the home. The largest potential source for home-based exposure was mercury spills from gas regulators. One publication estimated that mercury was spilled in 1,363 homes (Hryhorczuk et al. 2006). Although many children were likely exposed, information is not available to determine how many children were actually exposed in these 1,363 homes.

Although the ages of the children exposed ranged from 2 to 18 years, adolescent youths were the most frequent procurers of mercury. Depending upon clinical symptoms and the availability of laboratory tests, many of these children were tested for mercury exposure. The results ranged from < 0.20 to > 1,000 μg/L in urine and from < 4 to 295 μg/L in blood. Neither urine nor blood mercury levels correlate well with the presence or severity of symptoms (Cherry et al. 2002; Gattineni et al. 2007; Tominack et al. 2002).

### Exposure scenarios

Three location-based exposure scenarios broadly illustrate the nature and public health impact of mercury exposure events. The first two categories are scenarios in the home and at school, two common locations for childhood mercury exposures. The third category includes exposures at other locations, such as medical clinics and former industrial properties not adequately remediated.

#### Exposures at home

Although mercury-containing devices are becoming less common in the home, mercury is still found in some thermometers, barometers, thermostats, electric switches, natural gas regulators, and compact fluorescent lightbulbs (CFLs). Even the small amount of mercury in a typical thermometer (0.5–3.0 g or 0.04–0.22 mL) can be hazardous if spilled indoors and cleaned up improperly (Smart 1986; von Muhlendahl 1990). For example, a 9-year-old boy presented to a hospital with lethargy, limb pain, and unsteadiness (Rennie et al. 1999). The child’s physical examination showed mild facial weakness, areflexia, ataxia, and impaired sensation. An investigation revealed that 3 months earlier the boy had dismantled a mercury-containing sphygmomanometer in his bedroom. Sphygmomanometers contain approximately 11 mL of mercury (Caravati et al. 2008). On discovery of the spill, his parents had attempted to clean up the mercury by vacuuming. After diagnosis, the bedroom furniture was removed and a mercury vapor–absorbing filter system was used for 3 months to eliminate residual mercury vapor. The boy was treated, and his neurologic symptoms slowly resolved over 6 months.

Although less frequently reported, other sources of elemental mercury exposure have resulted in home-based exposures. Before 1961, residential natural gas meters and pressure regulators were placed inside the home in parts of the United States. Each gas regulator contained about 10 mL (~ 2 teaspoons) of mercury. As alternative methods became available to reduce gas pressure, the industry began placing regulators outdoors. As a result, gas utility companies started relocating indoor meters and pressure regulators outdoors. In 2000, a homeowner near Chicago, Illinois, discovered mercury in his basement after his meter and regulator were relocated. This homeowner called the regional poison control center, initiating what eventually became a multistate response to 500,000 potentially contaminated homes and businesses (Hryhorczuk et al. 2006).

Some folk healers recommend oral ingestion of mercury to treat *empacho* (indigestion). In addition, some practitioners of Caribbean and Latin American religions, such as voodoo, Santería, obeah, Palo, and Espiritismo, use mercury ceremonially (Johnson 1999; Newby et al. 2006; U.S. EPA 2002; Wendroff 2005; Zayas and Ozuah 1996). Mercury may be rubbed onto the skin, added to candles, or sprinkled around the home. These practices potentially expose practitioners and their families. Previous reports document the ceremonial mercury use in neighborhoods whose residents are largely Hispanic (John Snow Inc. 2003; Ozuah et al. 2003; Rogers et al. 2008, 2007; Zayas and Ozuah 1996). Because mercury contamination can persist for years, ceremonial mercury use in the home could also expose future occupants and their children, contributing to health disparities in these populations.

#### Exposures at school

The most common sources of mercury in schools are mercury stored in science laboratories, mercury in broken mercury-containing instruments, and mercury brought to school from other locations. Additionally, some gymnasium floors contain a mercury catalyst that releases mercury vapor into the air.

In 2004, 854 students at a middle school in Nevada were exposed to mercury (Azziz-Baumgartner et al. 2007; Burgess 2007). A student found a container of mercury in a storage shed and took it home. The student subsequently brought approximately 60 mL of the mercury to school, where several students played with it. Only 20 mL of the mercury brought to school was recovered. Mercury vapor levels inside the school reached 50 μg/m^3^. Of the 854 students potentially exposed, 200 completed an exposure history and provided urine samples. The mean urine mercury level for all students tested was within the normal range (mean = 0.36 μg/L; range, 0.14–11.4 μg/L). Students who reported having seen mercury (*n* = 66) had significantly higher urine mercury levels than those who did not. Those students who touched the mercury (*n* = 36) or got it on their clothes (*n* = 28) also had significantly higher urine mercury levels than those who did not. Few students had signs or symptoms of mercury toxicity.

From the 1960s to the 1980s, many schools installed synthetic gymnasium floors that contained a mercury catalyst in the polyurethane formulation for the floor covering; the finished product typically contained 0.1–0.2% mercury (ATSDR 2003). One manufacturer reported installing more than 25 million pounds of polyurethane flooring. These surfaces slowly release mercury vapor, particularly from damaged areas. The airborne mercury concentration in these gymnasiums varies from 0.79 to 1.6 μg/m^3^ (ATSDR 2003). In a similar report, mercury vapor measurements in the breathing zone were 0.042–0.050 μg/m^3^ (ATSDR 2004). The variation in airborne concentrations likely includes differences in sampling equipment, the size and condition of the floor, and indoor ventilation.

#### Exposures in other locations

Mercury exposures can also occur in medical facilities and in buildings where mercury was previously used. Sources include prior mercury spills, mercury stored on abandoned property, and mercury found in medical or dental offices. In some cases, mercury is carried or tracked to multiple locations, making the primary exposure location difficult to identify.

In most situations, the reuse of industrial property does not result in childhood mercury exposure. However, the trend toward redeveloping industrial property for other uses requires due diligence to ensure that past exposures do not become future health hazards. For example, in Hoboken, New Jersey, a building formerly used to manufacture mercury vapor lamps was converted to private condominiums (Orloff et al. 1997). After moving into the building, residents reported seeing mercury droplets on their oven and kitchen counters. Subsequent investigations revealed pools of mercury in the subflooring and elevated air mercury levels throughout the building. Urinary mercury concentrations of the occupants ranged from 4.8 to 133 μg/g creatinine. All occupants of the building were relocated, and the building was demolished.

Similarly, the New Jersey child care facility mentioned previously closed in 2006 after environmental sampling revealed mercury in dust (< 0.02–0.25 μg/wipe) and air (7.0–11.4 μg/m^3^) (ATSDR 2007b). After the facility closed, approximately one-third of the children were found to have urine mercury levels above the comparison value (5 μg/g creatinine). Serial testing confirmed that the elevated urine mercury levels decreased over time to below the comparison value.

Children may also be exposed to elemental mercury from abandoned industrial property. Two teenagers in Texarkana, Arkansas, removed a large amount of mercury from an abandoned neon sign plant (Lowry et al. 1999). The mercury weighed between 23 and 100 lb (770–3,300 mL). One teenager took mercury home and shared it with other children. Investigators found mercury contamination in 12 residences, a convenience store, and a school classroom. For persons who agreed to provide two rounds of urine and blood mercury tests, initial urine concentrations ranged as high as 68.7 μg/g creatinine, and blood mercury concentrations ranged as high as 104 μg/L. Neurobehavioral assessments of eight exposed individuals failed to establish a relationship between mercury exposure and test results.

Finally, mishandling of mercury and mercury-containing medical equipment occurs in some medical and dental offices. In one example, mercury was spilled from a sphygmomanometer (ATSDR 2001). A patient who observed the attempted cleanup called the poison control center. The state health department and the state EPA responded and measured breathing zone mercury levels between 45 and 50 μg/m^3^. Visible beads of mercury were observed in the clinic, which served both adults and children. Patients and staff were evacuated from the area, and a professional environmental contractor was hired to carry out remedial activities.

### Initiatives for preventing mercury exposure

In this review we focus on mercury exposures that are preventable, and several federal and state-based initiatives are designed to prevent future exposures. For example, in 2001 Congress passed the Small Business Liability Relief and Brownfields Revitalization Act, setting up the funding of grants for brownfield activities administered by the U.S. EPA. Brownfields are defined in the statute as “real property, the expansion, redevelopment, or reuse of which may be complicated by the presence or potential presence of a hazardous substance, pollutant, or contaminant” (Small Business Liability Relief and Brownfields Revitalization Act of 2001). The U.S. EPA Brownfields Program awards grants to state, tribal, and local governments and not-for-profit organizations to assess and clean up eligible brownfields, including sites that may have been contaminated with mercury through industrial activity or illegal disposal (U.S. EPA 2008a). States may oversee assessment and cleanup activities, where appropriate, to ensure the cleanup meets state standards.

Through its role in the brownfield initiative, ATSDR created a Brownfields/Land Reuse Steering Committee to assess the impacts of redevelopment on public health. This effort includes the broader health impacts of revitalization and a sustainable environment.

A few states have passed laws that affect locating schools and redeveloping property for use as a school. Ten states have laws that prohibit locating a school on or near pollution sources, including mercury-contaminated sites. Six states require environmental assessments for any new school locations. However, the vast majority of states have yet to adopt such regulations.

To reduce the amount of mercury entering the waste stream and lessen the incidence of spills and exposures, some states have restricted the sale and disposal of mercury-containing products. For example, legislation has been enacted (or proposed) regarding the sale or disposal of mercury-containing thermometers, thermostats, switches, relays, blood pressure devices, electronic appliances, batteries, and dental amalgams. Some legislation specifically targets the use of products containing mercury in schools or health care settings. The U.S. EPA provides a table of these initiatives by state on its Web site (U.S. EPA 2008). Currently, 45 states have various mercury initiatives.

Some states are developing initiatives to proactively educate teachers and students regarding the potential dangers of mercury exposures and to assist in school laboratory cleanouts. For example, the Illinois Department of Public Health has an interactive mercury education Web site that includes curricula for teachers, information on handling spills in the classroom, and activities for children to learn how to avoid exposure (Illinois Department of Public Health 2008).

### Limitations

Duplication and inconsistent reporting of events between data sources and within data sources make any estimate of the national incidence of mercury exposure to children unreliable. The quality and completeness of the information reported may be affected by personal liability for causing or cleaning up the spill. Spills in private residences are likely to be underreported because residents are unaware of the health hazard or the reporting requirements for certain mercury spills.

Published case reports and case series often provide exposure and health outcome information, but these are subject to reporting bias, retrospective data collection, and imprecise estimates of exposure dose and duration. In addition, published literature is likely to be biased toward reporting worst-case scenarios, as opposed to the more typical exposures that do not cause symptoms or attract attention. Despite their limitations, the data and literature reviewed here are the best available sources of information on children’s exposure to elemental mercury during release events in the United States.

## Discussion and Conclusions

For this review, we set out to address three objectives. Our first objective was to identify the common sources of elemental mercury exposure among children. We found that children are most frequently exposed to mercury when mercury is mishandled or when people improperly clean up spilled mercury. Children are exposed when mercury is scavenged, collected, and pooled from sources such as industrial property, school chemistry laboratories, and electrical or medical equipment (Azziz-Baumgartner et al. 2007; Baughman 2006; CDC 2005a, 2005b; Gordon 2004; Tominack et al. 2002). Providing locations to appropriately dispose of mercury and information on how to properly dispose of mercury can potentially reduce the likelihood that children will come in contact with stored mercury.

Broken thermometers are the most common exposure source, based on calls to AAPCC poison control centers. However, these calls are decreasing. This reduction may reflect the decreasing availability of mercury thermometers. In 2008, the Interstate Mercury Education and Reduction Clearinghouse of the Northeast Waste Management Officials’ Association reported an 11% decrease in mercury sales from 2001 to 2004 (Interstate Mercury Education and Reduction Clearinghouse 2008).

The second objective was to describe the location, demographics, and proportion of children affected by elemental mercury exposure in the United States. We divided mercury exposures into three categories based on location: in the home, at school, and at other locations, such as improperly remediated industrial property and medical facilities. At all locations, the primary exposure pathway of concern for elemental mercury is inhalation.

The demographics and proportion of U.S. children affected by these exposures are not directly quantifiable using the various data sources we reviewed. Most data sources that collect information on the release of hazardous substances do not systematically collect information on the persons affected. The typical exposure scenario involves relatively small amounts of mercury without reports of human illness. Neither urine nor blood mercury levels correlate well with the presence or severity of symptoms (Cherry et al. 2002; Gattineni et al. 2007; Tominack et al. 2002). Elevated mercury vapor levels have been documented, but demonstrable health effects are rarely reported after small mercury spills, such as broken fever thermometers. Regardless, all spills should be cleaned up properly.

## Recommendations

The third and final objective for this review was to clarify what is needed to reduce elemental mercury exposure and to appropriately respond to mercury spills and releases. The primary prevention guidelines below support the Healthy People 2010 goal to reduce human exposure to heavy metals such as mercury, as measured by blood and urine mercury concentrations (U.S. Department of Health and Human Services 2000).

### Health education

Some states have developed culturally appropriate educational materials on the risks of elemental mercury exposure and ways to prevent exposure. The federal government should encourage and support other states in developing similar materials. Persons that would benefit from these materials include parents, teachers, school administrators, children, medical and dental health care workers, religious practitioners, folk healers, and people who sell, develop, own, or manage real estate.

For parents, teachers, and school administrators, the educational messages should include health hazards of elemental mercury, sources of mercury exposure in homes and schools, risks associated with misuse and damage to mercury-containing devices, proper disposal of the mercury present in homes and schools, mercury substitutes and mercury-free devices, proper containment and cleanup procedures for small mercury spills, and sources of additional information on mercury exposure and related health effects.

For children, the educational messages should include sources of mercury exposure in homes and schools, how to identify mercury, health risks of playing with mercury, and what to do if mercury is found.

For health care practitioners, the educational messages should include importance of stopping exposure for everyone at risk; signs, symptoms, and health effects of acute and chronic mercury exposure; how to ask patients about mercury exposure; and resources available for information and medical consultation.

Public health professionals also should communicate with religious practitioners and botanica owners who use or sell mercury. The educational messages provided for parents, teachers, and children are also important for practitioners who use mercury ceremonially. Although no single predictable path to success exists, culturally sensitive communication is important to ensure that people who engage in these practices understand the acute and chronic health risks associated with mercury exposure.

Finally, persons involved in the management, redevelopment, sales, or leasing of industrial or residential property should be aware of mercury-related hazards, whom to contact if mercury is found, and the applicable state and local liability laws.

### Federal, state, and local policy initiatives

Some states have developed policy initiatives to reduce the potential for mercury exposure. For example, after the event at the New Jersey child care, the State of New Jersey passed a law requiring that an environmental evaluation be conducted before a child care or school is opened (ATSDR 2007b). The federal government should encourage and support other states in developing similar initiatives to reduce mercury exposures in their communities. Moreover, each initiative should include the ability to assess its effectiveness.

The primary prevention capacities recommended in the CDC’s *Preventing Lead Exposure in Young Children: A Housing-Based Approach to Primary Prevention of Lead Poisoning* (2004) comprise a framework for making housing lead-safe by preventing future exposures and protecting previously exposed children from further exposure. Although mercury exposures are less pervasive than are environmental lead exposures, some similarities exist between the two types of exposures, and these can assist in developing mercury-based prevention initiatives.

State and local mercury initiatives can assist in preventing mercury exposure by promoting alternatives, such as mercury-free devices and mercury substitutes; providing for safe and secure disposal of recovered mercury and mercury-containing products, including CFLs; providing information on the purchase of mercury cleanup kits; ensuring that land and buildings chosen for redevelopment undergo sufficient environmental review of previous activities; and determining whether property remediation is sufficiently protective for future occupants.

### Surveillance

Although small spills are often not reported, better surveillance of such low-risk exposures is not likely to protect children. The various data sources reviewed suggest that most releases do not lead to demonstrable harm if the exposure period is short and the mercury is properly cleaned up. Small spills of mercury (i.e., the quantities of mercury in fever thermometers or less) are easily handled by adults who are familiar with mercury cleanup procedures. Errors made in handling elemental mercury are best addressed through education and policy initiatives that preempt or minimize exposure potential.

Larger releases of mercury (i.e., more than the amount in a fever thermometer) cause greater concern. As the amount of mercury released increases, so does the risk of harmful exposure and subsequent health effects. More comprehensive information and longitudinal follow-up of persons exposed to larger spills or releases are needed.

This kind of follow-up requires enhanced coordination between environmental responders (e.g., U.S. EPA, U.S. Coast Guard National Response Center) and collectors and providers of exposure and health outcome information (e.g., AAPCC, ATSDR, CDC). Better coordination would increase the effectiveness of existing surveillance mechanisms by assembling more information on factors that can affect exposure level, such as the amount spilled, temperature, air flow, room volume and ventilation, exposure duration, exposure measuring instruments, methodology used to measure exposure, and types of cleanup methods employed. This information would then assist in interpreting the health impact of individual exposures, along with longitudinal clinical and laboratory data. With this information, federal and state health agencies can increase their understanding of children’s elemental mercury exposures and respond appropriately to this public health hazard.

## Figures and Tables

**Table 1:** Geometric means, selected percentiles, and the corresponding 95% CI for urine mercury concentrations (μg/L) for children sampled as part of NHANES, 2003–2004.

Age group (years)	Sample size (no.)	Geometric mean (95% CI)	Selected percentile (95% CI)			
			50th	75th	90th	95th
6–11	286	0.254 (0.213–0.304)	0.190 (0.160–0.230)	0.430 (0.330–0.560)	1.14 (0.610–1.61)	1.96 (1.13–2.97)
12–19	722	0.358 (0.313–0.408)	0.320 (0.270–0.360)	0.700 (0.530–0.840)	1.59 (1.13–2.52)	2.83 (1.88–3.66)

**Table 2:** Characteristics of HSEES-reported mercury events, 2002–2006.^a^

Event	No. (%)
Mercury events	843 (100)
Total events affecting children	409 (49)
State reporting event	
Reporting all 5 years	
Colorado	5 (1)
Iowa	8 (2)
Louisiana	0 (0)
Minnesota	56 (14)
New Jersey	73 (18)
New York	129 (32)
North Carolina	5 (1)
Oregon	4 (1)
Texas	6 (2)
Utah	19 (5)
Washington	7 (2)
Wisconsin	32 (8))
Reporting 4 years	
Missouri	39 (10)
Reporting 2 years	
Alabama	0 (0)
Florida	7 (2)
Michigan	16 (4)
Mississippi	3 (1)
Type of release	
Spill only	360 (88)
Volatilization	6 (2)
Spill and volatilization	40 (10)
Fire	1 (< 1)
Not reported	2 (< 1)
Location of event	
Private household	307 (75)
School	98 (24)
Other^b^	4 (1)
Contributing cause of event	
Equipment failure	27 (7)
Human error	357 (87)
Intentional or illegal release	18 (4)
Unknown	7 (2)

a Percentages may total > 100% because of rounding.

b Includes private property other than a home (3) and a restaurant (1).

**Table 3:** Mercury events reported to the U.S. Coast Guard National Response Center that potentially exposed children, by location, 2002–2007 (*n* = 113).

Category	No.^a^
School	50
Home	5
Medical facility or clinic	1
Other location^b^	14
Location not reported	45

a Exposure locations are not mutually exclusive; therefore, the number of locations does not total the number of reported events (*n* = 113). In addition, location is likely biased by the selection criteria of including all exposure events at schools or child care facilities.

b Category includes events with street addresses when the specific location (i.e., school or home) could not be determined.
